# Low Levels of Microbial Translocation Marker LBP Are Associated with Sustained Viral Response after Anti-HCV Treatment in HIV-1/HCV Co-Infected Patients

**DOI:** 10.1371/journal.pone.0118643

**Published:** 2015-03-18

**Authors:** Jessica Nyström, Jenny Stenkvist, Amanda Häggblom, Ola Weiland, Piotr Nowak

**Affiliations:** 1 Department of Laboratory Medicine, Division of Clinical Microbiology, Karolinska Institutet, Karolinska University Hospital, Huddinge, Stockholm, Sweden; 2 Unit of Infectious Diseases, Department of Medicine, Karolinska Institutet, Karolinska University Hospital, Huddinge, Stockholm, Sweden; 3 Department of Infectious Diseases, County Council of Gävleborg, Gävle, Sweden; Temple University School of Medicine, UNITED STATES

## Abstract

**Background:**

Microbial translocation (MT) contributes to immune activation during HIV and HCV infections. We investigated the kinetics of MT markers during anti-HCV and anti-HIV treatments, and if baseline plasma levels of lipopolysaccharide (LPS), lipopolysaccharide binding protein (LBP) and soluble CD14 (sCD14) could predict anti-HCV treatment outcome.

**Methods:**

Plasma from 78 HIV-infected patients was evaluated for LPS, LBP and sCD14. The patients starting anti-HCV treatment (with ongoing antiretroviral (ART) treatment) were categorized into sustained viral responders (SVR; n = 21) or non-responders (NR; n = 15) based on treatment outcome. ART starting subjects—were categorized into chronically HCV-infected (CH; n = 24) and mono-infected (HIV; n = 18), based on the HCV infection status. Samples were collected before start (at baseline) of pegylated-interferon-alpha/ribavirin (peg-IFN/RBV) or antiretroviral-therapy and two years after treatment start (at follow up). χ^2^–test, non-parametric statistics and logistic regression were applied to determine the associations with treatment response and changes of the soluble markers.

**Results:**

Plasma levels of LPS and sCD14 were elevated in all subjects before antiviral-treatment but remained unchanged at follow-up. Elevated levels of LBP were present in patients with HIV and HIV/HCV co-infection and were reduced by ART. Additionally, higher levels of LBP were present at baseline in NR vs. SVR. Higher levels of LBP at baseline were associated with non-response to peg-IFN/RBV treatment in both bivariate (OR: 0.19 95% CI: 0.06–0.31, p = 0.004) and multivariate analysis (OR: 1.43, 95% CI: 1.1–1.86, p = 0.07).

**Conclusion:**

In HIV/HCV co-infected patients high baseline LBP levels are associated with non-response to peg-IFN/RBV therapy. Plasma LBP (decreased by ART) may be a more relevant MT marker than LPS and sCD14.

## Introduction

Co-infection with hepatitis C virus (HCV) is common in patients with HIV-1 infection and HCV associated liver failure is a leading cause of death in HIV/HCV infected patients [1]. Several studies have shown that HIV infection promotes HCV hepatic fibrosis progression, even in subjects on effective antiretroviral treatment (ART) [2]. The pathological events during the co-infection are not fully revealed, however most relevant mechanisms include direct viral effect, dysregulation of cytokine network / inflammation, increased oxidative stress and hepatocyte apoptosis [3]. These processes lead to acceleration of liver inflammation giving rise to enhanced fibrosis progression in HIV/HCV co-infected individuals.

In recent years, systemic inflammation associated with gut derived microbial products has been described during both HIV-1 [4] and HCV infections [5]. Thus, HIV-1 causes a profound CD4^+^ T lymphocyte depletion in the gastrointestinal tract [6] leading to a structural defect in the gut-blood barrier that permits translocation of microbial products to the blood [4]. Increased levels of circulating lipopolysaccharide (LPS) are found in patients with HIV-1 infection and linked to immune activation by triggering monocyte activation via CD14 / TLR4 pathway and release of pro-inflammatory stimuli [7]. LPS also stimulates production of lipopolysaccharide binding protein (LBP) from e.g. hepatocytes, which in a feedback mechanism shuttles LPS to CD14 and exert pro-inflammatory signals or in excess binds and neutralizes LPS, diminishing inflammation [8].

Microbial products also play a substantial role in the development of liver fibrosis. LPS can stimulate liver resident Kupffer cells as well as hepatic stellate cells and promote fibrogenesis [9]. Data derived from animal models suggest that microbial products accelerate liver fibrosis both directly and by activation of the immune system [9, 10]. Moreover, elevated LPS levels have also been found during chronic HCV and HBV infections, non-alcoholic steatosis and are associated with severity of liver disease [11–14]. The role of microbial products in HIV/HCV co-infected individuals have been approached with reports showing elevated levels of LPS and sCD14 compared to mono-infected individuals [15, 16]. The HIV/HCV co-infected patients have a higher degree of hepatic fibrosis and anti-HCV treatment is less effective than in HCV mono-infected persons [1].

Understanding the role of MT in regulation of HIV/HCV co-infection may lead to development of therapeutic strategies for the management of the patients with ongoing liver disease. However, there is a lack of longitudinal studies on microbial translocation in HIV/HCV co-infection and therefore we aimed to investigate the kinetics of microbial translocation (MT) markers in HIV/HCV co-infected individuals. Additionally, we studied whether the baseline plasma levels of MT markers measured by LPS, LBP and sCD14 could predict the outcome of pegylated interferon and ribavirin treatment. Our results show an association of low levels of LBP with anti-HCV treatment response. Moreover, we describe the decline of LBP levels after ART.

## Materials and Methods

### Study design, participants, settings and eligibility

**Fig 1:**
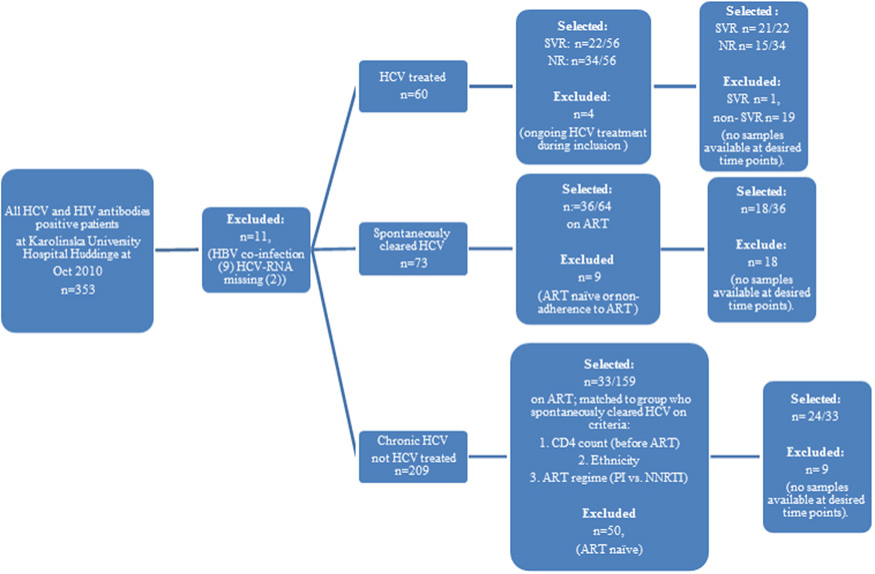
Cohort inclusion and exclusion criteria. Abbreviations: Sustained viral responders (SVR), Non-responders (NR), Human immunodeficiency virus type 1 (HIV-1), Hepatitis C virus (HCV), Hepatitis B virus (HBV), antiretroviral therapy (ART). Non-Nucleoside Reverse Transcriptase Inhibitors (NNRTIs), Protease Inhibitors (PIs).

We conducted a retrospective study in 78 patients. Patients were selected from the cohort of 353 anti-HCV and HIV seropositive individuals enrolled in clinical care at Department of Infectious Diseases, Karolinska University hospital, Huddinge between 1991–2011. Inclusion and exclusion criteria are presented in Fig. 1. Shortly, all patients’ alive in October 2011, age 18 or older were included. Exclusion criteria: HBV co-infection, clinically documented non-adherence to ART. The final selection was based upon the availability of stored plasma samples at selected time-points in the bio-bank.

Patients were categorised into four groups:

1. Non responders (NR) n = 15; HIV-1/ HCV co-infected patients with on-going ART which was initiated prior to treatment with pegylated interferon-alpha and ribavirin (peg-IFN/RBV). Lack of response was defined as HCV-RNA levels ≥ 50 IU/ml and <2 log 10 reduction from baseline after 24 weeks of treatment (full treatment course lasted 24 weeks for genotype 2–3 or 48 weeks for genotype 1 and 4). Samples were collected at start of HCV-treatment (baseline) defined as a time-point within six months before start of anti-HCV and at two years of follow up.
2. Sustained viral responders (SVR) n = 21: HIV-1/ HCV co-infected patients with on-going ART which was initiated prior to treatment with peg-IFN/RBV. Sustained viral response was defined as undetectable HCV-RNA (<50 IU/mL) 24 weeks after end of treatment (full treatment course lasted 24 weeks treatment for genotype 2–3 or 48 weeks for genotype 1 and 4). Samples were collected at start of HCV-treatment (baseline) defined as a time-point within six months before start of anti-HCV and at two years of follow up.
3. Chronically HCV infected (CH) n = 24; HIV-1/HCV co-infected patients without anti-HCV treatment but with ART. Samples were collected at start of ART-treatment (baseline) defined as a time-point within six months before start of ART and at two years of follow up.
4. HIV-1 mono-infected (HIV) n = 18: HIV-1 infected patients who had spontaneously cleared their HCV infection before introduction of ART (anti HCV antibodies positive, HCV RNA negative). Samples were collected at start of ART-treatment (baseline) defined as a time-point within six months before start of ART and at two years of follow up.

The patients in group 3 and 4 were matched for CD4 T-cell count before ART, ethnicity, ART-regime. The study was approved by the Regional ethics committee (Stockholm, 2010/1782–31/2) and followed the ethical guidelines of the Helsinki declaration. Due to the retrospective nature of our study, no written informed consent was given by participants; therefore patient records/information was anonymized and de-identified prior to analysis.

### Quantification of plasma LPS, lipopolysaccharide binding protein (LBP) and soluble CD14

LPS levels were quantified on stored (-80°C) plasma samples using the commercially available Limulus Amebocyte Assay (LAL assay, Lonza, Basel, Switzerland), as previously described [17]. Soluble CD14 (sCD14) and LBP levels in plasma were quantified by ELISA assay following manufacturer’s instructions (R&D systems, Minneapolis, USA and Hycult biotech, Uden, Netherlands) In all assays samples at baseline and follow-up from the same patient were assayed on the same 96 well plate.

### Viro-immune characteristics and estimation of liver disease

HIV RNA load, HCV RNA load and CD4+ and CD8^+^ T cell counts were measured as part of the clinical routine with Roche Taqman Real-Time PCR, Cobas Amplicor (Roche Molecular System Inc, Branchburg, New Jersey, USA) and flow cytometry, respectively. HCV genotyping was performed with a line probe assay (Inno-LiPA HCV II, Innogenetics NV, Gent, Belgium) or an in-house method [18, 19]. The aspartate aminotransferase (AST) and platelet levels were evaluated at the routine clinical chemistry laboratory at Karolinska University hospital at the sampling day. The values were incorporated at AST-to- platelet ratio index (APRI), which was calculated to estimate the stage of fibrosis [20]. APRI have aside from HCV also been repeatedly validated in HCV/HIV co-infection [21–23] and HIV- mono-infected [24]. APRI score < 0.5 indicated lack of significant fibrosis as suggested by Resino et al [25].

### Statistical analysis

Categorical variables are presented as number and percentage, subsequently analyzed with χ^2^-test. Numerical variables are presented as median with interquartile range (IQR). Wilcoxon matched-pairs signed rank test was used for assessing changes between baseline and post-intervention results. T-test or Mann Whitney U test was used to assess the differences between the groups at the same time point. Correlations of baseline parameters were analysed by Spearman´s rank test. P value < 0.05 was considered as significant. The analyses were performed using Graph Pad Prism v.5.04 software.

Bivariate logistic regression model was applied to identify if baseline variables were associated with NR or SVR. Odds ratios (ORs) and their 95% confidence intervals (CIs) were computed. Factors considered in the bivariate logistic regression model were: route of transmission, HCV genotype, type of ART, time on ART, IL28B genotype, Sweden as country of birth, age and sex, APRI- score at baseline, baseline levels of plasma markers (sCD14, LPS, LBP, CD4, CD8, HCV-RNA), and change in plasma markers from follow-up to baseline (Δ) (LBP, LPS, sCD14, CD4, CD8). Variables with a p-value close to or less than 0,2 in bivariate analysis or considered clinically relevant regardless of the p-value, were tested in a multivariate logistic regression model. The model selection was done with Akaike Information Criterion (AIC) [26]. Listweise deletion regression was preformed, but only one patient had a missing variable. The final logistic regression model was adjusted for HCV genotype IL28B and baseline LBP, adjusted odds ratios (aORs) and their 95% confidence intervals (CIs) were computed. Analyses were performed using Stata version 12.1 SE (Statacorp).

## Results

### Baseline characteristics

The patient’s baseline characteristics are presented in Table 1. As expected NR and SVR had significantly higher CD4+ T cell counts than the HIV and CH (p< 0.0001) at baseline, due to preceding ART. The initial CD4 +T cell counts difference warranted further comparison analysis within the anti-HCV and anti-HIV treatment groups.

**Table 1:** Baseline characteristics of study population.

Characteristics	ALL (78)	SVR (21)	NR (15)	SVR vs. NR (p-value)	HIV (18)	CH (24)	HIV vs. CH (p-value)
**Age, years**	46 (41–51)	49 (43–54)	48 (44–50)	0.56	44 (50–50)	45 (39–49)	
**Sex**							
**Male/female**	55/23	17/4	11/4		9/9	18/6	
**Duration of ART before HCV-treatment**	57 (31–112)	72 (47/147)	36 (16–96)	0.04	0	0	
**in month**							
**ART regim ^**A**^**							
**PI**	54 (69%)	13 (62%)	11 (73%)		12 (67%)	18 (75%)	
**NNRTI, NRTI, other combinations**	24 (31%)	8 (38%)	4 (27%)		6 (33%)	6 (25%)	
**Transmission**							
**MSM/bisexual**	6 (8%)	2 (9.5%)	3 (20%)		1 (6%)		
**IDUs**	54 (69%)	12 (57%)	9 (60%)		12 (66%)	21 (87.5%)	
**Heterosexual**	11 (14%)	1 (5%)	2 (13%)		5 (28%)	3 (12.5%)	
**Other**	7 (9%)	6 (28.5%)	1 (7%)				
**CD4 T cells/μL**	307 (210–470)	498 (384–619)	405 (360–640)	0.86	210 (161–283)	233 (150–280)	0.74
**CD4 T cells %**	19 (14–25)	25 (19–35)	24 (16–28)	0.32	18 (15–20)	14 (10–18)	0.04
**CD8 T cells/μL**	956 (574–1277)	1080 (658–1320)	1166 (610–1288)	0.97	598 (343–901)	995 (456–1483)	0.10
**SCD8 T cells %**	57 (50–64)	52 (45–58)	47 (38–57)	0.40	58 (53–66)	64 (59–75)	0.08
**Baseline HCV-RNA log 10 IU/ML**	6.3 (5.79–6.78)	5.9 (5.52–6.64)	6.3 (5.8–6.72–9)	0.35	0	6.7 (6.30–7.27)	
**Baseline HIV-RNA copies/ml**	5.0 (2.3–6.0)	<20	<20		5.0 (2.3–6.0)	5.0 (3.5–6.0)	0.47
**sCD14 ng/ml**	2544 (3013–3592)	3052 (2276–3868)	3056 (1933–3662)	0.35	2529 (2027–3407)	2272 (1843–3023)	0.23
**LPS pg/ml**	128 (116–147)	124 (113–151)	129 (114–167)	0.62	140 (120–151)	127 (108–139)	0.10
**LBP μg/ml**	16.60 (11–23)	11 (9–14)	22.6 (16–33)	0.001	14.25 (10–18)	23 (17–37)	0.0006
**HCV-genotype**							
**1 & 4**	34 (61%)	9 (43%)	12 (80%)			13 (65%)	
**2 & 3**	22 (39%)	12 (57%)	3 (20%)			7 (35%)	
**missing information**						4	
**APRI score**	0.64 (0.43–1.07)	0.8 (0.57–1.74)	0.89 (0.42–1.34)	0.22	0.37 (0.26–1.08)	0.59 (0.46–0.82)	0.43
**% available APRI score at Baseline**	68%	76%	87%		56%	58%	
**APRI score < 0,5**	32%	12%	38%		50%	36%	
**APRI score > 0,5**	68%	88%	62%		50%	64%	
**IL28B genotype**							
**CC**	34 (44%)	8 (38%)	4 (27%)		11 (61%)	11 (46%)	
**non-CC**	44 (56%)	13 (62%)	11 (73%)		7 (39%)	13 (54%)	

Data are presented as median (IQR) or percentage. Differences between groups were compared by Man Whitney U or χ^2^—test at baseline.

^A^ HIV and CH group initiated the ART-treatment at baseline, p≤ 0.05 was considered significant.

Abbreviations: SVR = Sustained viral responders, NR = non-responder, HIV = HIV-1 mono-infected, CH = Chronic HCV infected, ART = Antiretroviral therapy, NRTIs = Nucleoside reverse transcriptase Inhibitors, NNRTIs = Non-Nucleoside reverse transcriptase Inhibitors, PIs = Protease Inhibitors, MSM = men who have sex with men, IDUs = injection drug users, HCV = Hepatitis C virus, HIV = Human immunodeficiency virus. APRI = AST /platelet ratio index. LPS = lipopolysaccharide, LBP = lipopolysaccharide binding protein.

At baseline, no significant differences concerning CD4+ T cell counts, CD8+ T cell counts, HCV RNA plasma levels and APRI score were observed between SVR and NR. The preceding ART duration was significantly longer in the individuals with SVR compared to those with NR (p = 0.04). Baseline analysis of CH and HIV; groups which initiated ART did not reveal statistically significant differences as to CD4/CD8 T cell counts, HIV-RNA viral load, APRI score at the starting point.

### Immune status and APRI score after peg-IFN/RBV—treatment

**Fig 2:**
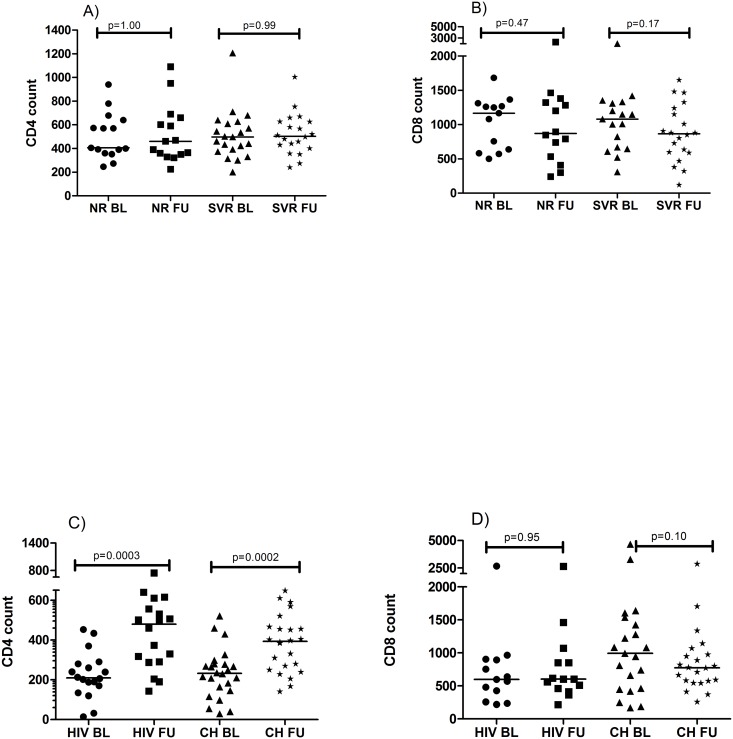
Kinetics of CD4 and CD8 T cell counts. Baseline (BL) and Follow-up (FU) of CD4 + T cell counts and CD8+ T cell counts during the study time were compared between Sustained viral responders (SVR) and non-responder (NR) group (A-B) and between HIV and HCV chronic (CH) group (C-D). Changes between baseline and follow-up were assessed by Wilcoxon matched-pairs signed rank-test; p≤ 0.05 was considered significant.

Two years after HCV-treatment, SVR and NR had similar CD4+ T cell levels as at baseline NR p = 1.0; SVR: p = 0.99 (Fig. 2A), with no intergroup differences concerning CD4 + T cell kinetics during study period (at baseline: p = 0.86; at follow-up: p = 0.64). The CD8+ T cell levels at follow-up were similar as at baseline; NR: p = 0.47; SVR: p = 0.17 (Fig. 2B). As expected, APRI score was significantly reduced after anti-HCV treatment in SVR compared to NR (Δ APRI, p = 0.006).

### Viro-immune status and liver characteristics after ART

ART resulted in a significant increase of CD4+T cell counts at follow-up in both HIV and CH as compared to baseline HIV: p = 0. 0003; CH: p< 0.0002, (Fig. 2C). Also HIV-RNA levels had significantly decreased in both groups after two years on ART, HIV: p = 0.0002 and CH: p< 0.0001 (data not shown). In details, two of twenty-four patients in CH-group hade detectable HIV-RNA levels at follow-up (100 and 509 copies/ml respectively) and five of eighteen in HIV-group had detectable HIV-RNA (range: 23–100 copies/ml). There was no intergroup difference concerning CD4+ T cells gain (baseline: p = 0.74, follow-up p = 0.31). The CD8+ T cell counts were not changed at follow-up HIV: p = 0.95; CH: p = 0.1 (Fig. 2D) and the CD8+ T cell kinetics were similar between HIV and CH (baseline: p = 0.10, follow-up p = 0.37). APRI score had significantly increased at follow-up in CH (p = 0.02), but remained unchanged in HIV (p = 0.63).

### Sustained LPS and sCD14 levels after peg-IFN/RBV-treatment

**Fig 3:**
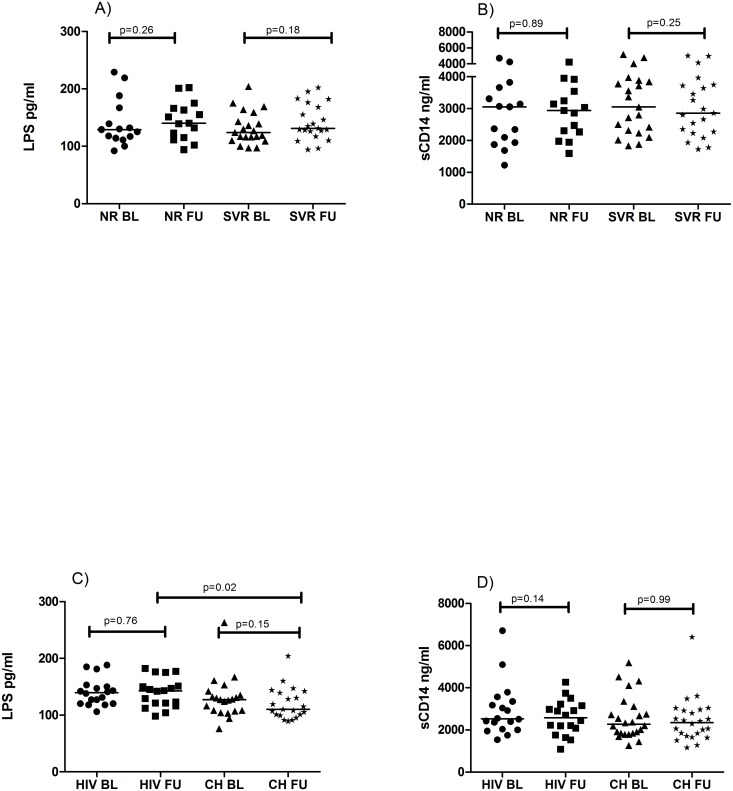
Kinetics of LPS and sCD14. A-D) Baseline (BL) and follow-up (FU) levels of LPS and sCD14 during the study time were compared between Sustained viral responders (SVR) and non-responder (NR) group (A-B) and between HIV and HCV chronic (CH) group (C-D). Wilcoxon matched-pairs signed rank test was used for assessing changes between baseline and follow-up. Mann Whitney U test was used to assess the differences between the groups at the same time point, p≤ 0.05 was considered significant.

In order to describe the kinetics of LPS and sCD14 during anti-HCV treatment, we compared these markers at baseline and follow-up. There were no significant differences in LPS levels at baseline between SVR vs. NR: p = 0.62 (Table 1) and the levels remained similar at two years of follow-up in both groups NR: p = 0.26; SVR: p = 0.18 (Fig. 3A). Additionally, there was no difference in LPS kinetics between the groups at follow-up (p = 0.77). NR and SVR had similar sCD14 levels at baseline p = 0.35 (Table 1) and both groups had sustained sCD14 levels at follow-up NR: p = 0.89; SVR: p = 0.25 (Fig. 3B). Moreover, there was no significant correlation between LPS nor sCD14 and CD4 or CD8 levels at baseline or follow-up. LPS and sCD14 where not interrelated at any of the time points.

### Unchanged LPS and sCD14 levels after ART

Plasma LPS levels were similar before introduction of ART in HIV and CH p = 0.10 (Table 1) but at follow-up CH had significant lower levels of LPS compared to the HIV p = 0.02 (Fig. 3C). LPS kinetics were similar in the two patient groups, HIV: p = 0.76; CH: p = 0.15 (Fig. 3C). At baseline the sCD14 levels were akin in HIV and CH p = 0.23 (Table 1) and did not change after two years of ART, HIV: p = 0.14; CH: p = 0.99 (Fig. 3D).

### Kinetics of LBP

**Fig 4:**
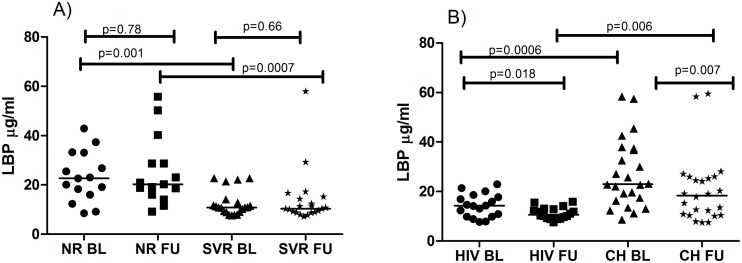
Kinetics of LBP. Baseline (BL) and follow-up (FU) levels of LBP levels during the study time was compared between Sustained viral responders (SVR) and non-responder (NR) group (A) and between HIV and HCV chronic (CH) group (B). Baseline and follow-up changes in LBP levels were assessed by Wilcoxon matched-pairs signed rank test. Mann Whitney U test was used to assess the differences between the groups at the same time point; p≤ 0.05 was considered significant.

Additionally, LBP was measured at baseline and follow-up in all patients. NR had significantly higher LBP levels than SVR at baseline p = 0.001 (Table 1) which remained higher at follow-up, p = 0.0007 (Fig. 4A). There was no difference between the kinetics of LBP in these two groups (Fig. 4A). Also CH had significantly higher LBP levels than HIV at baseline p = 0.0006 (Table 1) and this difference was also present at follow-up p = 0.006 (Fig. 4B). We found that plasma LBP levels at follow-up decreased significantly compared to baseline in HIV p = 0.018 and CH p = 0.007 (Fig. 4B). In the whole cohort a weak negative correlation was seen between baseline LBP and CD4 (r = -0.243, p = 0.032) and LBP and CD4% (r = -0.249, p = 0.028).

### Identification of factors associated to SVR or NR after peg-IFN/RBV treatment

To investigate the possible predictive value of microbial translocation on response to anti-HCV therapy, we conducted a bivariate and multivariate logistic regression analysis including HIV-1 and HCV related variables as well as LPS, LBP and sCD14 (Table 2). In the bivariate model, high plasma LBP levels at baseline were associated with an increased risk of non-response (OR: 0.19, 95% CI: 0.06–0.31, p = 0.004, Table 2). High LBP levels were also associated with non-response in the multivariate model (OR: 1.43, 95%CI; 1.1–1.86, p = 0.007, Table 2). HCV genotype (1 and 4) was also associated with non-response to anti-HCV treatment in both the bivariate (OR: 4.89, 95% CI: 1.05–22.84, p = 0.04, Table 2) and the multivariable model (OR: 244.52 95%CI; 2.51–23815.41, p = 0,019, Table 2).

**Table 2:** Bivariate and multivariable Logistic regression for Non-response to HCV treatment.

NR vs. SVR	Bivariate		Adjusted**	
	OR (95% CI)	p-value	aOR (95% CI)	p-value
**IDU**	1			
**Other**	0.89(0.23; 3.42)	0.864		
**Male**	1			
**Female**	1.55(0.32; 7.5)	0.589		
**Other**	1			
**Sweden**	1.08(0.16; 7.44)	0.935		
**Non-PI**	1			
**PI**	1.69(0.4; 7.17)	0.475		
**Time on ART**	0.99 (0.97–1.00)	0.086		
**Genotype 2&3**	1		1	
**Genotype 1&4**	4.89(1.05; 22.84)	0.044	244.52 (2.51; 23815.41)	0.019
**C/C**	1		1	
**Non C/C**	1.69(0.4; 7.17)	0.475	45.45 (0.67; 3104.02)	0.077
**Age at baseline**	0.98(0.9; 1.07)	0.662		
**APRI Baseline**	0.57(0.23; 1.43)	0.233		
**CD4 Baseline**	1(1; 1)	0.89		
**CD8 Baseline**	1(1; 1)	0.988		
**CD4/CD8 ratio**	1.03(0.03; 40.07)	0.986		
**Log10 HCV RNA**	1.9(0.62; 5.8)	0.258	13.01 (0.85; 199.51)	0.065
**LPS Baseline**	1.01(0.99; 1.03)	0.442		
**sCD14 Baseline**	1(1; 1)	0.354		
**LBP Baseline**	0.19(0.06;0. 31)	0.004	1.43 (1.1; 1.86)	0.007
Δ CD4*	1(1; 1.01)	0.756		
Δ CD8*	1(1; 1)	0.791		
Δ LPS*	0.99(0.96; 1.02)	0.488		
Δ sCD14*	1(1; 1)	0.59		
Δ LBP*	0(-0.06;0. 07)	0.936		

Bivariate and multivariate logistic regression analysis was performed including HIV-1 and HCV related variables as well as LPS, sCD14, LBP.

*Change from baseline (BL) to follow-up (FU),

**Logistic regression adjusted by LBP at baseline, HCV genotype, IL28B and Log10 HCV RNA at baseline. Model selection was done with AIC. Odds ratios (OR), adjusted OR (aOR) with 95% confidence interval (95% CI) and p-value are presented in the table. A p-value ≤ 0.05 was considered significant.

## Discussion

The main findings of our study were: a) the levels of all MT markers were elevated at baseline; b) the plasma levels of LBP were reduced by ART, while LPS and sCD14 remained unchanged during anti-HCV treatment and ART; c) low levels of LBP were predictive of virological response to peg-IFN/RBV treatment in HIV/HCV co-infected patients.

There is an increasing evidence that MT and MT-driven immune activation are important pathogenic mechanisms of hepatic disease in HCV mono-infection [5] as well as HIV/HCV co-infection [27]. In vitro data supported by animal studies show that microbial products (like LPS, CpG DNA) can promote both inflammation and fibrogenesis in the liver [9, 10]. Moreover increased levels of microbial products are found in the systemic circulation in patients with hepatic decompensation regardless of origin [11]. In general liver fibrosis/cirrhosis leading to hepatomegaly and increased portal hypertension causes translocation that is exacerbated by altered hepatic architecture, intestinal edema and dysfunction of Kupffer cells [28]. Clinically, spontaneous bacterial peritonitis (especially associated with Gram-negative bacteria) is not an uncommon event in patients with advanced liver cirrhosis [29]. Furthermore, hepatic encephalopathy is linked to endotoxemia and TLR4 single nucleotide polymorphism predicts the risk of liver cirrhosis in patients with chronic HCV [30].

We hypothesized that the levels of microbial products (as measured by LPS) and inflammatory response to microbial products (sCD14, LBP) are altered and elevated in patients with chronic HIV/HCV co-infection. Additionally we hypothesized that degree of MT is diminished after two years of anti-HCV and ART. Here, we present that levels of LPS, sCD14 and LBP were elevated at baseline in the whole cohort compared to previously published studies using the same methodology [5, 15, 17]. However, we could not observe a decrease of LPS and sCD14 two years after the initiation of effective ART [31, 32]. Additionally we could not see differences in the kinetics of these MT markers between the groups of patients during antiviral treatment. Earlier data have suggested that the LPS and sCD14 levels decrease with successful ART as MT and immune activation (IA) decreases [17, 33], however some reports have presented contradictory results with unchanged or increased levels of LPS/sCD14 after ART [31, 32]. The kinetics of sCD14 and LPS has not been extensively studied in the context of HIV/HCV co-infection and anti-HCV treatment. Published reports on microbial translocation in HCV and HBV mono-infected individuals showed elevated levels of LPS and sCD14 [5, 34], and association with liver fibrosis [35, 36]. Sandler e*t al*, did not find any significant changes in levels of sCD14 and LPS in the peg-IFN/RBV treated subjects with HCV or HBV (mono) infection [5] while Jirillo *et al* reported LPS decrease after IFN-α treatment in chronic HCV patients, even if sCD14 remained unchanged [37]. Additionally, Caradonna *et al* [38] showed association of lower levels of LPS with response to IFN/RBV. On the contrary Anthony *et al*, found elevated levels of sCD14 at 0.5 year after peg-IFN/RBV treatment in HIV/HCV co-infected patients [39].

The differences between these studies might be explained by heterogeneity of the cohorts, time of follow-up and sampling as well as contamination issues (in case of LPS). Additionally, detection of LPS with LAL-bioassay has high variability and the LPS values vary from study to study [40, 41]. sCD14 is a polyclonal marker in nature shedded from monocytes (but also e.g. neutrophils) not only by LPS stimulation but also by other bacterial and viral antigens [42]. Both markers are affected by sampling procedures or processing conditions e.g. food intake is known to affect the levels [43]. These factors were not controlled in our or other studies that have retrospective design.

The analysis of LBP levels at baseline showed higher levels in NR as compared to SVR as well as in CH as compared to HIV. This difference remained after two years of follow-up, although ART but not anti-HCV treatment caused substantial reduction of LBP in both HIV and CH. This drop in LBP was unified between the two ART-treated groups. Additionally low LBP levels were associated with SVR in both bivariate and multivariate models. Others have measured LBP showing elevated levels in co-infected (HIV/HCV) individuals compared to mono-infected (HIV or HCV) but also a lack of difference between those groups [16, 44]. We show clearly that levels of LBP are elevated in all patients with intergroup differences. Our findings of declining LPB levels after ART and association of low levels with treatment response are novel and to our knowledge have not been presented before. In general LBP seems to be a stable plasma marker that has a longer plasma half-life than LPS and may be more relevant to describe exposure to systemic lipopolysaccharide [45].

LBP is mainly synthesized in the liver; induced and released as a type-1 acute phase protein [46] by IL-1, IL-6 [46, 47] and also LPS from the microbiota, as well as non-infectious agents [48]. Among the LPS recognition molecules, LBP has the unique ability to recognize LPS multimers formed immediately after LPS is release to the bloodstream. LBP monomerize LPS and only this monomer can be recognized by other ‘‘LPS acceptors” like CD14, MD-2 and TLR-4 [49, 50]. The data suggests that LBP in a low concentration bind LPS and deliver it to the CD14/TLR4 receptor complex promoting the production of pro-inflammatory stimuli [8]. In excess the LBP binds LPS and neutralize it by shuttling it to the lipids promoting its elimination [8]. This self-limiting mechanism is of extreme importance for regulation of conditions associated with endotoxemia/ septicemia, however LBP may also play a role in metabolic syndrome [51]. As LBP is produced by hepatocytes one would expect that changes of LBP synthesis would occur with progressed liver fibrosis. This was not reflected by LBP levels in our cohort, as reported by others [52]. The baseline differences of LBP levels (especially NR vs. SVR) cannot solely be explained by effect of HIV and support that HCV infection plays a substantial role in the induction of pro-inflammatory cytokines and LBP. Most likely the origin is multifactorial as LBP can be trigged from hepatocytes, and monocytes/ macrophages not only by the translocated LPS originated from microbiota but also by proinflammatory cytokines like IL-1 and IL-6 which are dysregulated in both HIV and HCV infections. We believe that the reduction of LBP after effective ART is due to diminished inflammation and partially restored gut-blood barrier and as suggested by literature [53–55].

The evaluation of liver disease during the study time showed that APRI score substantially decreased in SVR patients at follow-up confirming the effectiveness of anti-HCV treatment. In ART treated subjects increased APRI score was noted in CH vs. HIV. The obvious question is why expected changes of liver status were not followed by changes in MT and MT associated immune activation. Other reports have established relationship between MT and progression of liver disease [5, 16, 56] and lower baseline levels of sCD14 have been associated with virological response to peg-IFN/RBV therapy [5, 39, 57]. However most of the data are derived from cross-sectional studies not following the longitudinal kinetics of the markers as presented here. This is why the causality of these findings cannot be excluded. We did not observe LPS or sCD14 changes during the anti-HCV nor ART-treatment. Most likely, HIV-HCV co-infected patients have a complex interplay between the HIV and HCV viruses that cannot be described by linear associations. In the HIV/HCV associated liver disease, microbial translocation is probably both a cause and effect of hepatic decompensation, leading to a vicious cycle of disease progression as suggested earlier [16, 27]. Additionally, as cells residing in the liver (hepatocytes/kupffer cells) contribute to production of sCD14 and LBP the associations between the plasma levels of above markers and fibrosis / liver dysfunction status should further be studied. Currently established factors associated with response to peg-IFN/RBV therapy for HCV infection include HCV genotype, age, race, HCV plasma levels and IL28B gene locus polymorphism [58]. Mechanisms underlying these associations are largely unidentified, although the integrity of the host immune system likely contributes. Our data confirm importance of HCV genotypes but we also add to these markers low LBP levels at baseline that were associated with therapy response to peg-IFN/RBV, both in the bivariate and multivariate analyses.

We acknowledge some limitations of our study. We focused on microbial translocation in HIV/HCV co-infected patients however; a control group of HCV mono-infected patients would have helped to clarify the relationship between the host response to microbial translocation, disease progression and treatment response. Additionally, the sample size of the cohort is limited and the information concerning clinical symptoms (e.g. diarrhoea) was unavailable in this retrospective study. In our work, we focused on direct MT-marker- LPS and sCD14 /LBP as measures of indirect response to microbial products. Inclusion of additional microbial indicators of MT would have been desirable though the uncertainty range of available markers make this doubtful [59].

We are aware that since introduction of a new HCV treatment, the INF-RBV treatment is not the standard of care anymore in the high income countries. Nevertheless the treatment is the most economic option in the low income countries. Additionally our work links phenomenon of microbial translocation with the HIV/HCV co-infection, elucidating the additional pathway for the therapeutic intervention.

In summary, our report shows complex interplay between the markers of microbial translocation /immune activation in patients with HIV/HCV co-infection. We could show that the markers of MT are elevated in patients but not affected by anti-HCV treatment. The association of LBP and SVR indicates that larger kinetics studies should be performed.
